# Whole-exome sequencing identifies mutations in *MYMK* in a mild form of Carey-Fineman-Ziter syndrome

**DOI:** 10.1212/NXG.0000000000000226

**Published:** 2018-03-19

**Authors:** Hadil Alrohaif, Ana Töpf, Teresinha Evangelista, Monkol Lek, Daniel McArthur, Hanns Lochmüller

**Affiliations:** From the MRC Centre for Neuromuscular Diseases (H.A., A.T., T.E., H.L.), Institute of Genetic Medicine, Newcastle University, England. Dr. Lochmüller is now with Department of Neuropediatrics and Muscle Disorders, Medical Center–University of Freiburg, Faculty of Medicine, Germany; Analytic and Translational Genetics Unit (M.L., D.M.), Massachusetts General Hospital, Boston; and Program in Medical and Population Genetics (M.L., D.M.), Broad Institute of Harvard and MIT, Cambridge, MA.

Fusion of single-nucleated myoblasts is essential for the formation of multinucleated myocytes. Mechanisms that regulate myoblast fusion have been a focus of recent studies.^1–4^ Transmembrane protein 8 (TMEM8C), also known as myomaker, is a highly conserved muscle-specific transmembrane protein encoded by the *MYMK* gene. The protein is expressed during early muscle development. *Mymk*-null mice die soon after birth because of skeletal muscle deficiency. In these mice, skeletal muscle tissue is present but consists of a smaller number of mononucleated cells indicating failure of myoblast cell fusion.^1^ Myomaker is also expressed during muscle regeneration when it coordinates fusion of satellite cells with residual muscle fibers to regenerate the damaged muscle tissue. In the absence of myomaker, adult mouse muscle tissue is unable to regenerate.^5^

In humans, mutations in the *MYMK* gene have recently been described in 8 individuals (aged 7–37 years) from 3 families with Carey-Fineman-Ziter syndrome (CFZS), a syndrome encompassing a congenital myopathy with marked facial weakness and Pierre Robin sequence, among other consistent features.^6^

Here, we report an additional and the oldest known patient-bearing mutations in the *MYMK* gene, identified through whole-exome sequencing (WES). We provide insights into disease progression, as well as ascertain features associated with the disorder.

## Clinical description

The patient is a 69-year-old British white man with juvenile-onset proximal myopathy. Distal muscles were also affected, although to a lesser extent. Weakness is mild and symmetrical with muscle power in the range of 3–4/5 for proximal muscles and 4–5/5 in distal ones. The patient also had marked facial weakness, lagophthalmos, minimal limitations in horizontal gaze, dysphagia, and chronic gastrointestinal (GI) symptoms. He reports alternating diarrhea and constipation that have been attributed to irritable bowel syndrome. The patient recalls weakness from his late teens when he was not as able as his peers. He particularly recalls being unable to climb ropes or blow balloons. His symptoms were first brought to medical attention at the age of 19 years when he dislocated his right knee and was noted to have muscle weakness. Progression of the myopathy has not been remarkable, and he remains ambulant, with the main complaint being the GI symptoms that are associated with food avoidance and weight loss.

**Figure 1:**
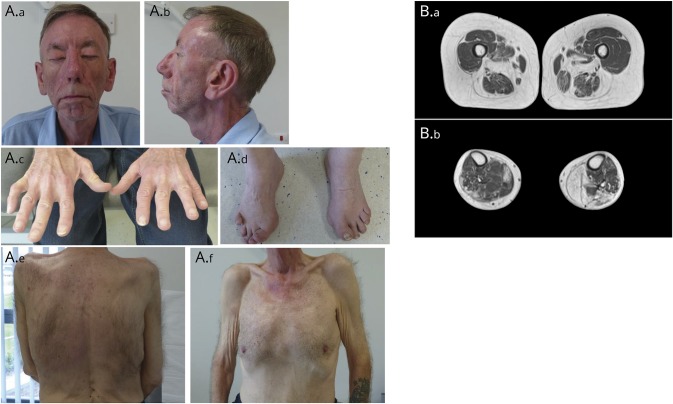
Clinical features and muscle MRI in *MYMK*-related Carey-Fineman-Ziter syndrome (A.a and A.b) Front and profile facial photographs demonstrating lagophthalmos (A.a, patient attempting lid closure), muscle hypoplasia, retrognathia, and broad nasal tip. (A.c and A.d) Wasting of intrinsic hand muscles and contracture deformities of the right little finger and the toes. (A.e, A.f) Scoliosis (A.e) and generalized muscle atrophy with pectoralis muscle hypoplasia (A.f). (B) T2-weighted MRIs of the thighs (B.a) showing severe fatty replacement of hamstrings, thigh adductors, and sartorius muscles, with relative sparing of the gracilis and quadriceps muscles bilaterally, and of the calves (B.b) showing asymmetric involvement with more marked fatty replacement in muscle of the right leg. Gastrocnemius and soleus muscles are severely affected, and the tibialis anterior on the right is relatively spared.

He had no cardiac or respiratory involvement, and his cognition was intact. The patient had mild dysmorphic features in the form of micrognathia, high-arched palate, and a prominent broad nasal tip (figure 1A). He also had spinal rigidity, scoliosis, bilateral pectoralis hypoplasia, and cryptorchidism. He also developed epilepsy, sensorineural hearing loss, unilateral cataracts, and glaucoma.

**Figure 2:**
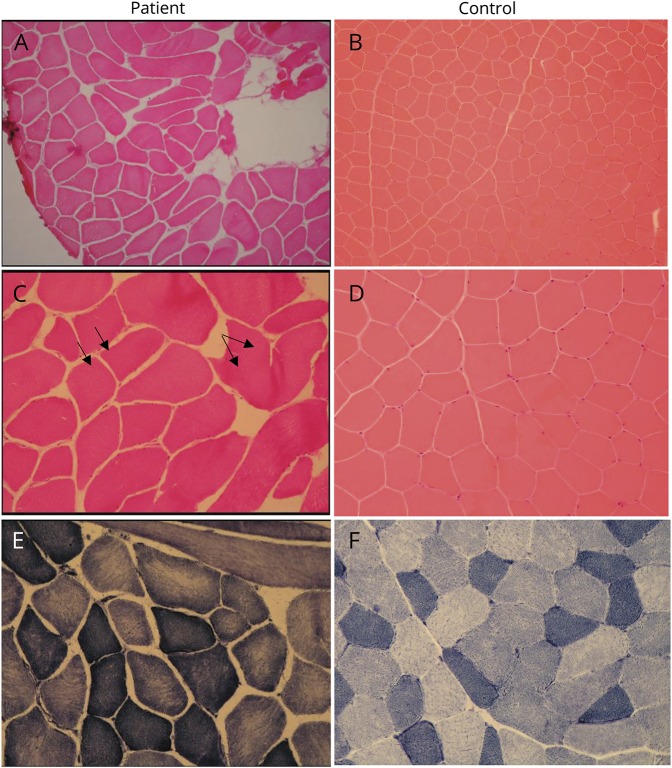
Needle biopsy of the left vastus lateralis Patient images and control images. H&E stain demonstrates fiber-size variation (A and B; H&E ×100) and occasional internal nuclei (C and D; H&E ×200, arrows). Mild moth-eaten changes seen on nicotinamide adenine dinucleotide (NADH) stain, indicating uneven mitochondrial enzyme activity within the sarcoplasm (E and F; NADH ×200).

Serum creatinine kinase levels were mildly elevated (500–1,000 IU/L), and EMG showed a picture suggestive of a chronic mildly active necrotizing myopathy. MRI of his lower limbs showed selective and asymmetric involvement (figure 1B). Muscle biopsy showed nonspecific myopathic features, namely, fiber-size variation and occasional central nuclei (figure 2, A-F).

## Genetic study

WES was performed at the Broad Institute of Harvard and MIT's Genomics Platform (Cambridge, MA) using >250 ng DNA (>2 ng/μL) in a 38-Mb targeted Illumina exome capture. Data were then analyzed on the Seqr interface (seqr.broadinstitute.org), initially using a candidate gene approach, consisting of a panel of 416 genes (musclegenetable.fr/, July 2016) known to be implicated in neuromuscular disorders, then searching for variants across the whole exome. This identified 2 heterozygous variants in the *MYMK* gene: c.271C>A (p.Pro91Thr) and c.553T>C (p.Cys185Arg). Both variants were previously reported as disease-causing mutations.^6^

## Discussion

Our patient presented with a mild slow progressing myopathy. Extraskeletal muscle manifestations pointed toward a syndromic myopathy rather than an isolated muscle disease. At the age of 69 years, he remains ambulant and shows slow progression of weakness. He shows no cardiac or respiratory involvement. The patient has a mild form of CFZS associated with *MYMK* mutations. Mainly proximal myopathy places *MYMK*-associated CFZS in the differential diagnosis for the limb-girdle muscular dystrophies. Pierre Robin sequence and cryptorchidism (in males) are consistent features described in association with *MYMK* mutations, and both may be a consequence of muscle dysfunction in early development. A recent report associated CFZS with mutations in the *STAC3* gene; a t-tubule protein involved in excitation-contraction coupling. Distinguishing features of *STAC3*-CFZS are short stature and malignant hyperthermia.^7^ Whether other clinical features present in our patient are a consequence of *MYMK* mutations remains to be established through continued follow-up of the known patients^6^ and diagnosis and follow-up of new patients with CFZS.
